# Extirpation of Splenic Fever by Agricultural Regulations

**Published:** 1898-01

**Authors:** 


Extirpation of Splenic Fever by Agricultural Regulations.. By Lydtin.—It is always encouraging to hear the veterinarian praised on the part of agricultural associations. For this reason the report which comes from the last session of the Agricultural Council for Alsace-Lorraine is of great interest. By doing away with the open pasturing of cattle splenic fever in the community of Illhausern has been very nearly suppressed. Formerly wild animals helped to spread the disease, and especially the carcass itself, which was only covered with dirt and often came to lie in surface-water; likewise flies and midgets carried the germs of the disease from such decaying carcasses. The district veterinarian Schield, of Rappoltsweiler, therefore deserves great credit for suppressing splenic fever by doing away with open pasturing in spite of popular opposition and of heavy immediate loss to his profession. His service, however, is to-day generally recognized, and he has saved the Government much money. We congratulate him on this event.—*Idem.*

---

### SELECTIONS.

---

Milk a Vehicle for Microbes; Needed Legislation.—The number of deaths caused annually by diseased milk and milk-contamination is sufficiently great to warrant legislative interference. The following points should be noted:

1. The need for setting up some authority, central or county, having in its charge the dairy farms and milk-shops of the country or county, with adequate power of inspection and regulation of all premises on which milk is produced, manipulated, or sold.

2. Statutory inspection by medical officers of health of all such premises in their districts, with right of entry at all reasonable hours and on all occasions, with power of inspection of all cattle, apparatus, utensils, etc.

3. Power to local authority immediately to prohibit the sale of milk reported by their medical officer of health to be causing, in his opinion, disease in human consumers, the dairymen, etc., interested having the onus of showing within twenty four hours valid reason why the prohibition should be removed, and the producing of reasons to call for reimbursement of the amount of loss sustained by the action of the local authority.

4. Certain diseases (to be defined and, if need be, added to) of teats and udders of milch-cows to be scheduled as infectious diseases of cattle for the purpose of securing penalty on all persons selling milk secreted by cattle so suffering.

5. All persons keeping milch-cows, or selling milk, to seek registration under local authority, under pain of heavy penalty for neglect of this duty.

6. All such persons to be under obligations to furnish the local authority, at intervals, with complete or supplementary or amended lists of customers, wholesale or retail, at a specific rate of payment.

7. The cubic space per cow to be fixed, a minimum being 800 feet.

8. The manufacture of ice-cream to be placed on a basis of safety to its consumers, in respect of production, materials, place of manipulation, storage, etc.

9. No milk to be allowed to be used for food from a shed in which a cow is housed while suffering under a scheduled disease, even though the cow producing the milk be not herself visibly so suffering.—*British Medical Journal.*

---

THE Annual Convention of the National Master-shoers' Protective Association was a complete success. The St. Louis Association of Veterinarians joined in the festivities of the craft.

Rabies and Texas-fever are among the reported diseases in New York during September. Drs. W. H. Kelly, of Albany, and V. A. Moore, of Ithaca, were directed to make the necessary investigation.

